# 1-Methyl-2-[(*E*)-2,4,5-trimeth­oxy­styr­yl]­pyridinium benzene­sulfonate mono­hydrate

**DOI:** 10.1107/S1600536812007805

**Published:** 2012-02-29

**Authors:** Hoong-Kun Fun, Suchada Chantrapromma, Pumsak Ruanwas, Teerasak Anantapong, Nawong Boonnak

**Affiliations:** a X-ray Crystallography Unit, School of Physics, Universiti Sains Malaysia, 11800 USM, Penang, Malaysia; b Crystal Materials Research Unit, Department of Chemistry, Faculty of Science, Prince of Songkla University, Hat-Yai, Songkhla 90112, Thailand; c Department of Biotechnology, Faculty of Agro-Industry, Prince of Songkla University, Hat-Yai, Songkhla 90112, Thailand

## Abstract

The asymmetric unit of the title compound, C_17_H_20_NO_3_^+^·C_6_H_5_O_3_S^−^·H_2_O, comprises two 1-methyl-2-[(*E*)-2,4,5-trimeth­oxy­styr­yl]pyridinium cations, two benzene­sulfonate anions and two water mol­ecules. The cations exist in the *E* conformation with respect to the C=C bond; one cation is essentially planar while the other is slightly twisted, the dihedral angles between the pyridinium and phenyl rings being 1.23 (14) and 6.64 (13)°, respectively. In the crystal, cations, anions and water mol­ecules are linked by O—H⋯O hydrogen bonds and weak C—H⋯O inter­actions into chains along the *b* axis. π–π inter­actions with centroid–centroid distances in the range 3.5557 (16)–3.6876 (16) Å are observed. C—H⋯π inter­actions and a C⋯O short contact [2.94 (4) Å] are also observed.

## Related literature

For bond-length data, see: Allen *et al.* (1987 ▶). For background to, and applications of, hy­droxy­lated stilbenes, see: Elmali *et al.* (2006 ▶); Kimura (2005 ▶); Ko *et al.* (1999 ▶); Nitta *et al.* (2002 ▶); Olas & Wachowicz (2002 ▶); Park *et al.* (2008 ▶); Roupe *et al.* (2006 ▶); Son *et al.* (2007 ▶). For related structures, see: Chanawanno *et al.* (2010 ▶); Fun *et al.* (2011 ▶); Mueangkeaw *et al.* (2010 ▶). For the stability of the temperature controller used in the data collection, see: Cosier & Glazer (1986 ▶).
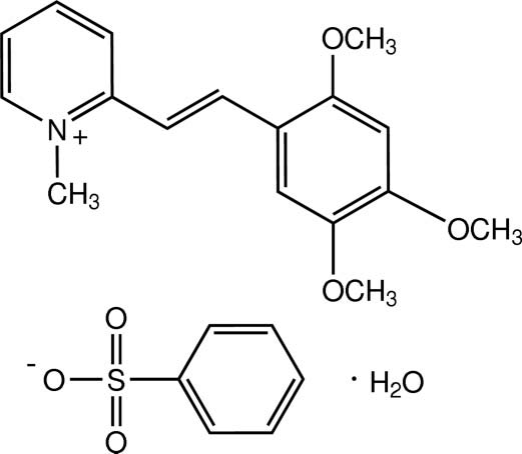


## Experimental

### 

#### Crystal data

C_17_H_20_NO_3_^+^·C_6_H_5_O_3_S^−^·H_2_O
*M* *_r_* = 461.53
Triclinic, 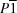

*a* = 11.2458 (4) Å
*b* = 13.5995 (5) Å
*c* = 15.3604 (6) Å
α = 73.345 (2)°
β = 84.623 (2)°
γ = 82.842 (2)°
*V* = 2228.92 (15) Å^3^
*Z* = 4
Mo *K*α radiation
μ = 0.19 mm^−1^
*T* = 100 K
0.44 × 0.22 × 0.16 mm

#### Data collection

Bruker APEXII CCD area-detector diffractometer
Absorption correction: multi-scan (*SADABS*; Bruker, 2005 ▶) *T*_min_ = 0.920, *T*_max_ = 0.970
38826 measured reflections
10142 independent reflections
6560 reflections with *I* > 2σ(*I*)
*R*_int_ = 0.057

#### Refinement

*R*[*F* ^2^ > 2σ(*F* ^2^)] = 0.066
*wR*(*F* ^2^) = 0.180
*S* = 1.03
10142 reflections
598 parameters
H-atom parameters constrained
Δρ_max_ = 0.72 e Å^−3^
Δρ_min_ = −0.45 e Å^−3^

### 

Data collection: *APEX2* (Bruker, 2005 ▶); cell refinement: *SAINT* (Bruker, 2005 ▶); data reduction: *SAINT*; program(s) used to solve structure: *SHELXTL* (Sheldrick, 2008 ▶); program(s) used to refine structure: *SHELXTL*; molecular graphics: *SHELXTL*; software used to prepare material for publication: *SHELXTL* and *PLATON* (Spek, 2009 ▶).

## Supplementary Material

Crystal structure: contains datablock(s) global, I. DOI: 10.1107/S1600536812007805/ez2283sup1.cif

Structure factors: contains datablock(s) I. DOI: 10.1107/S1600536812007805/ez2283Isup2.hkl

Supplementary material file. DOI: 10.1107/S1600536812007805/ez2283Isup3.cml

Additional supplementary materials:  crystallographic information; 3D view; checkCIF report

## Figures and Tables

**Table 1:** Hydrogen-bond geometry (Å, °) *Cg*5 and *Cg*6 are the centroids of the C18*A*–C23*A* and C18*B*–C23*B* rings, respectively.

*D*—H⋯*A*	*D*—H	H⋯*A*	*D*⋯*A*	*D*—H⋯*A*
O1*W*—H1*W*1⋯O5*B*	1.07	1.88	2.919 (3)	161
O1*W*—H2*W*1⋯O6*A*	0.99	1.87	2.843 (3)	169
O2*W*—H1*W*2⋯O5*A*^i^	0.93	1.98	2.894 (3)	169
O2*W*—H2*W*2⋯O4*B*	0.94	1.97	2.884 (4)	163
C1*A*—H1*AA*⋯O4*A*^ii^	0.93	2.24	3.159 (4)	169
C4*A*—H4*AA*⋯O6*A*	0.93	2.51	3.394 (3)	160
C1*B*—H1*BA*⋯O6*B*^iii^	0.93	2.34	3.216 (4)	157
C4*B*—H4*BA*⋯O4*B*^iv^	0.93	2.39	3.198 (3)	145
C6*B*—H6*BA*⋯O5*B*^v^	0.93	2.54	3.419 (4)	158
C14*B*—H14*D*⋯O6*B*^iii^	0.96	2.39	3.307 (4)	160
C14*B*—H14*F*⋯O5*B*^v^	0.96	2.51	3.331 (4)	143
C19*A*—H19*A*⋯O1*W*	0.93	2.59	3.477 (4)	160
C20*A*—H20*A*⋯O2*B*^vi^	0.93	2.60	3.393 (5)	144
C3*A*—H3*AA*⋯*Cg*5	0.93	2.78	3.688 (3)	164
C16*A*—H16*C*⋯*Cg*6^iv^	0.96	2.79	3.453 (4)	127

Symmetry codes: (i) 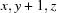; (ii) 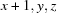; (iii) 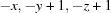; (iv) 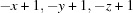; (v) 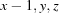; (vi) 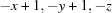.

**Fig. 1.**
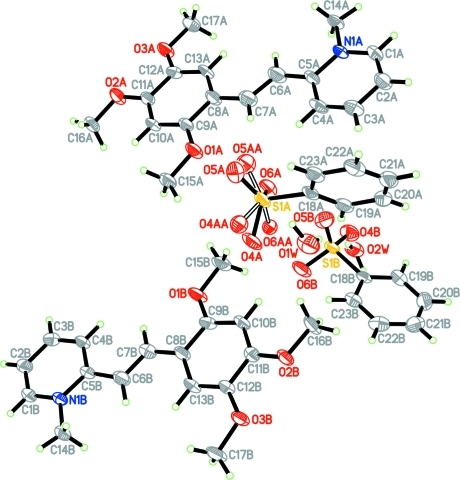
The molecular structure of the title compound, with 50% probability displacement ellipsoids and the atom-numbering scheme.

**Fig. 2.**
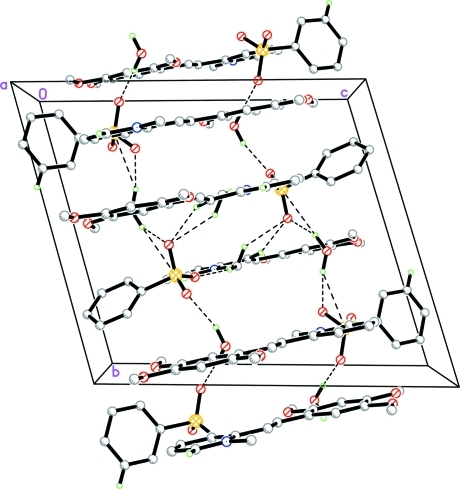
The crystal packing of the title compound viewed approximately down the a-axis. Only the major component is shown. For clarity, only H atoms involved in hydrogen bonds are shown. Hydrogen bonds are shown as dashed lines.

## supplementary crystallographic information

### Comment

Hydroxylated stilbenes are widely found in nature and also show interesting biological activities (Ko *et al.*, 1999; Park *et al.*, 2008; Roupe *et al.*, 2006; Son *et al.*, 2007). Resveratrol, a well known hydroxylated stilbene, is an isolated bioactive substance found in grapes and red wine (Kimura, 2005). It has shown various bioactivities such as antibacterial (Nitta *et al.*, 2002), antiplasmodial (Son *et al.*, 2007), antioxidant (Olas & Wachowicz 2002), anti-inflammatory (Elmali *et al.*, 2006) and anticancer activities (Kimura, 2005). Due to these activities, it led us to synthesize the hydroxylated pyridinium stilbenes and to evaluate their antimicrobial activity. The title compound (I) is a hydroxylated pyridinium stilbene derivative which was synthesized and tested for antibacterial activity against the *Bacillus subtilis*, *Enterococcus faecalis*, *Staphylococcus aureus*, Methicillin-Resistant *Staphylococcus aureus*, Vancomycin-Resistant *Enterococcus faecalis*, *Pseudomonas aeruginosa*, *Salmonella typhi* and *Shigella sonnei*, but unfortunately it was found to be inactive. Herein we report the crystal structure of (I).

Fig. 1 shows the asymmetric unit of (I) which consists of two C_17_H_20_NO_3_^+^ cations, two C_7_H_7_O_4_S^-^ anions and two solvent water molecules. The SO_3_ moiety of one anion (molecule *A*) is disordered over two positions with the refined site-occupancy ratio of 0.948 (4):0.052 (4). The cations exist in the *E* configuration with respect to the C6═C7 double bonds [1.358 (4) Å in molecule *A* and 1.352 (4) Å molecule *B* ] with the torsion angle C5–C6–C7–C8 = -179.4 (3)° in molecule *A* [175.8 (3)° in molecule *B*]. One cation is essentially planar (molecule *A*) whereas the other (molecule *B*) is slightly twisted with the dihedral angle between the pyridinium and phenyl rings being 1.23 (14) and 6.64 (13)°, respectively. Three methoxy substituent groups of the 2,4,5-trimethoxyphenyl are almost co-planar with their attached phenyl ring with torsion angles of C15–O1–C9–C10, C16–O2–C11–C12 and C17–O3–C12–C13 being 5.2 (4), 173.3 (3) and 0.4 (4)° respectively in molecule *A* [the corresponding values are -2.0 (4), 178.6 (2) and 4.9 (4) ° in molecule *B*]. These angle values also indicated that the *para* methoxy group (at atom C11) points toward the *ortho* methoxy group (at atom C9) whereas it points away from the *meta* methoxy group (at atom C12) (Fig. 1) due to the steric effect of their positions. The two anions are inclined with respect to the two cations in which the C18A–C23A benzene ring of the anion makes dihedral angles of 81.28 (14) and 79.93 (14)° with the N1A/C1A–C5A and N1B/C1B–C5B pyridinium rings, respectively and the C18B–C23B benzene ring makes dihedral angles of 36.38 (14) and 34.35 (14)° with the N1A/C1A–C5A and N1B/C1B–C5B pyridinium rings. The bond lengths of (I) are in normal ranges (Allen *et al.*, 1987) and are comparable to those in related structures (Fun *et al.*, 2011; Mueangkeaw *et al.*, 2010).

In the crystal packing (Fig. 2) O—H···O hydrogen bonds and weak C—H···O interactions (Table 1) link cations, anions and water molecules into chains along the *b* axis. π–π interactions were observed with the distances of Cg_1_···Cg_2_^vi^ = 3.6780 (17) Å (symmetry code (vi) = 2-*-x*, -*y*, 1-*z*), Cg_1_···Cg_4_^i^ = 3.5951 (17) Å, Cg_2_···Cg_3_^i^ = 3.5557 (16) Å and Cg_3_···Cg_4_^ii^ = 3.6876 (16) Å; Cg_1_, Cg_2_, Cg_3_ and Cg_4_ are centroids of N1A/C1A–C5A, C8A–C13A, N1B/C1B–C5B and C8B–C13B rings, respectively. A C16A···O4AA^vii^ [2.94 (4) Å; (vii) = 1-*-x*, -*y*, 1-*z*] short contact was observed. The crystal is stabilized by weak C—H···O, C—H···π (Table 1) and π–π interactions.

### Experimental

The title compound was synthesized by mixing a 1:1 molar ratio of 1-methyl-2-[(*E*)-2,4,5-trimethoxystyryl]pyridinium iodide (0.10 g, 0.24 mmol) which was prepared according to the previous method (Mueangkeaw *et al.*, 2010) and silver (I) benzenesulfonate (Chanawanno *et al.*, 2010) (0.06 g, 0.24 mmol) in methanol (50 ml). The mixture immediately yielded a grey precipitate of silver iodide. After stirring the mixture for ca. 30 min, the precipitate of silver iodide was removed and the resulting solution was evaporated yielding the title compound as an orange solid. Orange block-shaped single crystals of (I) suitable for *x*-ray structure determination were recrystallized from DMSO by slow evaporation at room temperature over a few weeks, Mp. 455-456 K.

### Refinement

All H atoms were positioned geometrically and allowed to ride on their parent atoms, with d(O-H) = 0.92 - 1.07 Å, d(C-H) = 0.93 Å for aromatic and CH and 0.96 Å for CH_3_ atoms. The *U*_iso_ values were constrained to be 1.5*U*_eq_ of the carrier atom for methyl H atoms and 1.2*U*_eq_ for the remaining H atoms. A rotating group model was used for the methyl groups. The SO_3_group of one benzenesulfonate is disordered over two positions with the refined site-occupancy ratio of 0.948 (4):0.052 (4).

### Crystal data

**Table:**

C_17_H_20_NO_3_^+^·C_6_H_5_O_3_S^−^·H_2_O	*Z* = 4
*M**_r_* = 461.53	*F*(000) = 976
Triclinic, *P*1	*D*_x_ = 1.375 Mg m^−^^3^
Hall symbol: -P 1	Melting point = 455–456 K
*a* = 11.2458 (4) Å	Mo *K*α radiation, λ = 0.71073 Å
*b* = 13.5995 (5) Å	Cell parameters from 10142 reflections
*c* = 15.3604 (6) Å	θ = 2.2–27.5°
α = 73.345 (2)°	µ = 0.19 mm^−^^1^
β = 84.623 (2)°	*T* = 100 K
γ = 82.842 (2)°	Block, orange
*V* = 2228.92 (15) Å^3^	0.44 × 0.22 × 0.16 mm

### Data collection

**Table:**

Bruker APEXII CCD area-detector diffractometer	10142 independent reflections
Radiation source: sealed tube	6560 reflections with *I* > 2σ(*I*)
Graphite monochromator	*R*_int_ = 0.057
φ and ω scans	θ_max_ = 27.5°, θ_min_ = 2.2°
Absorption correction: multi-scan (*SADABS*; Bruker, 2005)	*h* = −14→14
*T*_min_ = 0.920, *T*_max_ = 0.970	*k* = −17→17
38826 measured reflections	*l* = −19→19

### Refinement

**Table:**

Refinement on *F*^2^	Primary atom site location: structure-invariant direct methods
Least-squares matrix: full	Secondary atom site location: difference Fourier map
*R*[*F*^2^ > 2σ(*F*^2^)] = 0.066	Hydrogen site location: inferred from neighbouring sites
*wR*(*F*^2^) = 0.180	H-atom parameters constrained
*S* = 1.03	*w* = 1/[σ^2^(*F*_o_^2^) + (0.0791*P*)^2^ + 1.8818*P*] where *P* = (*F*_o_^2^ + 2*F*_c_^2^)/3
10142 reflections	(Δ/σ)_max_ = 0.001
598 parameters	Δρ_max_ = 0.72 e Å^−^^3^
0 restraints	Δρ_min_ = −0.45 e Å^−^^3^

### Special details

**Table:**

Experimental. The crystal was placed in the cold stream of an Oxford Cryosystems Cobra open-flow nitrogen cryostat (Cosier & Glazer, 1986) operating at 100.0 (1) K.
Geometry. All esds (except the esd in the dihedral angle between two l.s. planes) are estimated using the full covariance matrix. The cell esds are taken into account individually in the estimation of esds in distances, angles and torsion angles; correlations between esds in cell parameters are only used when they are defined by crystal symmetry. An approximate (isotropic) treatment of cell esds is used for estimating esds involving l.s. planes.
Refinement. Refinement of F^2^ against ALL reflections. The weighted R-factor wR and goodness of fit S are based on F^2^, conventional R-factors R are based on F, with F set to zero for negative F^2^. The threshold expression of F^2^ > 2sigma(F^2^) is used only for calculating R-factors(gt) etc. and is not relevant to the choice of reflections for refinement. R-factors based on F^2^ are statistically about twice as large as those based on F, and R- factors based on ALL data will be even larger.

### Fractional atomic coordinates and isotropic or equivalent isotropic displacement parameters (Å^2^)

**Table:**

	*x*	*y*	*z*	*U*_iso_*/*U*_eq_	Occ. (<1)
O1A	0.64507 (17)	0.11731 (17)	0.51621 (14)	0.0336 (5)	
O2A	0.69138 (17)	0.04628 (17)	0.83918 (14)	0.0327 (5)	
O3A	0.91709 (17)	0.06224 (17)	0.81037 (14)	0.0314 (5)	
N1A	1.15749 (19)	0.16459 (17)	0.32746 (16)	0.0220 (5)	
C1A	1.1978 (3)	0.1857 (2)	0.2393 (2)	0.0290 (7)	
H1AA	1.2795	0.1901	0.2246	0.035*	
C2A	1.1220 (3)	0.2008 (3)	0.1707 (2)	0.0335 (7)	
H2AA	1.1512	0.2154	0.1101	0.040*	
C3A	1.0001 (3)	0.1937 (2)	0.1943 (2)	0.0341 (7)	
H3AA	0.9465	0.2026	0.1494	0.041*	
C4A	0.9595 (2)	0.1734 (2)	0.2840 (2)	0.0291 (7)	
H4AA	0.8779	0.1695	0.2992	0.035*	
C5A	1.0373 (2)	0.1587 (2)	0.3530 (2)	0.0247 (6)	
C6A	0.9983 (3)	0.1373 (3)	0.4472 (2)	0.0362 (8)	
H6AA	1.0555	0.1282	0.4896	0.043*	
C7A	0.8816 (3)	0.1295 (2)	0.4777 (2)	0.0351 (7)	
H7AA	0.8263	0.1382	0.4341	0.042*	
C8A	0.8348 (3)	0.1090 (2)	0.5718 (2)	0.0297 (7)	
C9A	0.7104 (2)	0.1028 (2)	0.59021 (19)	0.0252 (6)	
C10A	0.6599 (2)	0.0813 (2)	0.67848 (19)	0.0244 (6)	
H10A	0.5778	0.0763	0.6895	0.029*	
C11A	0.7317 (2)	0.0672 (2)	0.75031 (19)	0.0241 (6)	
C12A	0.8564 (2)	0.0750 (2)	0.73381 (19)	0.0241 (6)	
C13A	0.9063 (2)	0.0947 (2)	0.6466 (2)	0.0275 (7)	
H13A	0.9886	0.0987	0.6362	0.033*	
C14A	1.2469 (2)	0.1503 (2)	0.3959 (2)	0.0272 (7)	
H14A	1.3256	0.1562	0.3659	0.041*	
H14B	1.2446	0.0832	0.4385	0.041*	
H14C	1.2286	0.2022	0.4276	0.041*	
C15A	0.5171 (3)	0.1185 (3)	0.5306 (2)	0.0331 (7)	
H15A	0.4818	0.1344	0.4730	0.050*	
H15B	0.4865	0.1698	0.5610	0.050*	
H15C	0.4972	0.0519	0.5674	0.050*	
C16A	0.5639 (3)	0.0492 (3)	0.8575 (2)	0.0336 (7)	
H16A	0.5454	0.0371	0.9218	0.050*	
H16B	0.5344	−0.0033	0.8373	0.050*	
H16C	0.5266	0.1156	0.8258	0.050*	
C17A	1.0434 (3)	0.0711 (3)	0.7967 (2)	0.0334 (7)	
H17A	1.0760	0.0636	0.8541	0.050*	
H17B	1.0570	0.1376	0.7562	0.050*	
H17C	1.0819	0.0181	0.7707	0.050*	
S1A	0.59991 (6)	0.15568 (6)	0.23898 (5)	0.02814 (19)	
O4A	0.47821 (19)	0.1975 (2)	0.21697 (16)	0.0399 (7)	0.948 (4)
O5A	0.6119 (2)	0.04475 (18)	0.28039 (16)	0.0383 (7)	0.948 (4)
O6A	0.65514 (19)	0.21139 (17)	0.29030 (15)	0.0328 (6)	0.948 (4)
O4AA	0.488 (3)	0.113 (3)	0.221 (3)	0.028 (10)*	0.052 (4)
O5AA	0.665 (4)	0.099 (3)	0.313 (3)	0.036 (12)*	0.052 (4)
O6AA	0.558 (3)	0.262 (3)	0.245 (2)	0.021 (9)*	0.052 (4)
C18A	0.6856 (2)	0.1748 (2)	0.1330 (2)	0.0250 (6)	
C19A	0.7215 (2)	0.2718 (2)	0.0885 (2)	0.0290 (7)	
H19A	0.6977	0.3272	0.1123	0.035*	
C20A	0.7934 (3)	0.2851 (3)	0.0081 (2)	0.0335 (7)	
H20A	0.8191	0.3494	−0.0214	0.040*	
C21A	0.8267 (3)	0.2032 (3)	−0.0279 (2)	0.0356 (8)	
H21A	0.8757	0.2124	−0.0812	0.043*	
C22A	0.7880 (3)	0.1078 (3)	0.0144 (2)	0.0347 (7)	
H22A	0.8091	0.0533	−0.0111	0.042*	
C23A	0.7177 (2)	0.0935 (2)	0.0952 (2)	0.0304 (7)	
H23A	0.6919	0.0291	0.1241	0.036*	
O1B	0.28112 (17)	0.39190 (17)	0.41584 (14)	0.0330 (5)	
O2B	0.25360 (17)	0.45714 (16)	0.09077 (14)	0.0303 (5)	
O3B	0.02436 (17)	0.47482 (17)	0.11018 (14)	0.0304 (5)	
N1B	−0.2427 (2)	0.35988 (18)	0.58282 (16)	0.0244 (5)	
C1B	−0.2907 (3)	0.3367 (2)	0.6690 (2)	0.0306 (7)	
H1BA	−0.3726	0.3304	0.6795	0.037*	
C2B	−0.2229 (3)	0.3223 (2)	0.7410 (2)	0.0317 (7)	
H2BA	−0.2574	0.3066	0.8001	0.038*	
C3B	−0.1002 (3)	0.3315 (2)	0.7243 (2)	0.0280 (7)	
H3BA	−0.0518	0.3228	0.7724	0.034*	
C4B	−0.0510 (2)	0.3535 (2)	0.6363 (2)	0.0265 (6)	
H4BA	0.0311	0.3586	0.6255	0.032*	
C5B	−0.1224 (2)	0.3684 (2)	0.5628 (2)	0.0246 (6)	
C6B	−0.0768 (3)	0.3963 (2)	0.4691 (2)	0.0328 (7)	
H6BA	−0.1313	0.4195	0.4238	0.039*	
C7B	0.0418 (3)	0.3905 (2)	0.4439 (2)	0.0300 (7)	
H7BA	0.0944	0.3715	0.4905	0.036*	
C8B	0.0947 (2)	0.4104 (2)	0.3527 (2)	0.0272 (6)	
C9B	0.2210 (2)	0.4087 (2)	0.3390 (2)	0.0262 (6)	
C10B	0.2777 (2)	0.4236 (2)	0.2528 (2)	0.0255 (6)	
H10B	0.3610	0.4201	0.2451	0.031*	
C11B	0.2087 (2)	0.4438 (2)	0.17786 (19)	0.0239 (6)	
C12B	0.0817 (2)	0.4511 (2)	0.18900 (19)	0.0239 (6)	
C13B	0.0278 (2)	0.4331 (2)	0.2751 (2)	0.0273 (7)	
H13B	−0.0555	0.4359	0.2825	0.033*	
C14B	−0.3247 (3)	0.3742 (3)	0.5090 (2)	0.0344 (7)	
H14D	−0.4046	0.3629	0.5350	0.052*	
H14E	−0.2981	0.3259	0.4746	0.052*	
H14F	−0.3246	0.4432	0.4696	0.052*	
C15B	0.4093 (3)	0.3863 (3)	0.4067 (2)	0.0378 (8)	
H15D	0.4404	0.3683	0.4659	0.057*	
H15E	0.4335	0.4521	0.3716	0.057*	
H15F	0.4399	0.3348	0.3764	0.057*	
C16B	0.3813 (3)	0.4530 (3)	0.0747 (2)	0.0348 (7)	
H16D	0.4017	0.4661	0.0106	0.052*	
H16E	0.4172	0.3858	0.1062	0.052*	
H16F	0.4107	0.5042	0.0967	0.052*	
C17B	−0.1048 (3)	0.4897 (3)	0.1180 (2)	0.0365 (8)	
H17D	−0.1351	0.5106	0.0583	0.055*	
H17E	−0.1300	0.5423	0.1482	0.055*	
H17F	−0.1353	0.4263	0.1526	0.055*	
S1B	0.68516 (6)	0.65230 (6)	0.30445 (5)	0.02695 (19)	
O4B	0.77426 (19)	0.71188 (18)	0.32296 (16)	0.0391 (6)	
O5B	0.7331 (2)	0.54663 (17)	0.31094 (15)	0.0401 (6)	
O6B	0.57193 (19)	0.6606 (2)	0.35600 (15)	0.0437 (6)	
C18B	0.6532 (2)	0.7081 (2)	0.1882 (2)	0.0249 (6)	
C19B	0.7214 (3)	0.7823 (2)	0.1321 (2)	0.0306 (7)	
H19B	0.7838	0.8042	0.1551	0.037*	
C20B	0.6959 (3)	0.8236 (2)	0.0415 (2)	0.0354 (8)	
H20B	0.7413	0.8737	0.0037	0.042*	
C21B	0.6034 (3)	0.7910 (2)	0.0068 (2)	0.0362 (8)	
H21B	0.5877	0.8179	−0.0543	0.043*	
C22B	0.5352 (3)	0.7188 (2)	0.0632 (2)	0.0354 (8)	
H22B	0.4719	0.6979	0.0403	0.043*	
C23B	0.5593 (3)	0.6767 (2)	0.1536 (2)	0.0296 (7)	
H23B	0.5128	0.6275	0.1912	0.035*	
O1W	0.6043 (2)	0.42912 (18)	0.22536 (18)	0.0468 (6)	
H1W1	0.6480	0.4851	0.2442	0.070*	
H2W1	0.6213	0.3548	0.2557	0.070*	
O2W	0.77573 (19)	0.88811 (19)	0.39160 (16)	0.0427 (6)	
H1W2	0.7312	0.9433	0.3541	0.064*	
H2W2	0.7657	0.8252	0.3799	0.064*	

### Atomic displacement parameters (Å^2^)

**Table:**

	*U*^11^	*U*^22^	*U*^33^	*U*^12^	*U*^13^	*U*^23^
O1A	0.0137 (10)	0.0592 (15)	0.0269 (12)	−0.0028 (9)	−0.0017 (9)	−0.0104 (10)
O2A	0.0171 (10)	0.0534 (14)	0.0271 (12)	−0.0036 (9)	0.0021 (9)	−0.0116 (10)
O3A	0.0152 (10)	0.0482 (13)	0.0315 (12)	−0.0024 (9)	−0.0026 (9)	−0.0124 (10)
N1A	0.0116 (11)	0.0283 (13)	0.0278 (13)	−0.0014 (9)	−0.0013 (9)	−0.0108 (10)
C1A	0.0155 (14)	0.0363 (17)	0.0338 (18)	−0.0023 (12)	0.0013 (12)	−0.0085 (13)
C2A	0.0217 (16)	0.048 (2)	0.0299 (17)	−0.0001 (14)	0.0000 (13)	−0.0118 (14)
C3A	0.0200 (15)	0.0418 (19)	0.042 (2)	−0.0007 (13)	−0.0065 (14)	−0.0134 (15)
C4A	0.0113 (13)	0.0309 (16)	0.048 (2)	−0.0026 (11)	−0.0018 (13)	−0.0151 (14)
C5A	0.0136 (13)	0.0267 (15)	0.0339 (17)	−0.0002 (11)	0.0028 (12)	−0.0105 (12)
C6A	0.0216 (16)	0.051 (2)	0.0359 (19)	0.0008 (14)	−0.0031 (14)	−0.0124 (15)
C7A	0.0245 (16)	0.0383 (18)	0.042 (2)	−0.0007 (13)	−0.0031 (14)	−0.0109 (15)
C8A	0.0156 (14)	0.0431 (18)	0.0293 (17)	−0.0003 (12)	0.0017 (12)	−0.0105 (14)
C9A	0.0148 (14)	0.0338 (16)	0.0250 (16)	0.0005 (11)	−0.0014 (12)	−0.0066 (12)
C10A	0.0109 (13)	0.0313 (16)	0.0311 (16)	−0.0005 (11)	0.0007 (11)	−0.0101 (12)
C11A	0.0168 (14)	0.0291 (15)	0.0258 (16)	0.0000 (11)	0.0007 (12)	−0.0084 (12)
C12A	0.0165 (14)	0.0294 (16)	0.0276 (16)	0.0021 (11)	−0.0046 (12)	−0.0108 (12)
C13A	0.0099 (13)	0.0369 (17)	0.0361 (18)	−0.0007 (11)	0.0009 (12)	−0.0121 (13)
C14A	0.0117 (13)	0.0373 (17)	0.0337 (17)	−0.0022 (11)	−0.0025 (12)	−0.0116 (13)
C15A	0.0161 (15)	0.047 (2)	0.0345 (18)	−0.0027 (13)	−0.0033 (13)	−0.0081 (15)
C16A	0.0179 (15)	0.049 (2)	0.0298 (17)	−0.0017 (13)	0.0061 (13)	−0.0072 (14)
C17A	0.0192 (15)	0.0418 (19)	0.0412 (19)	−0.0030 (13)	−0.0063 (13)	−0.0136 (15)
S1A	0.0132 (3)	0.0387 (4)	0.0323 (4)	−0.0053 (3)	0.0019 (3)	−0.0095 (3)
O4A	0.0122 (11)	0.0612 (19)	0.0437 (15)	−0.0014 (10)	−0.0004 (10)	−0.0118 (12)
O5A	0.0338 (14)	0.0379 (14)	0.0404 (14)	−0.0093 (11)	0.0037 (11)	−0.0058 (11)
O6A	0.0225 (12)	0.0429 (14)	0.0344 (13)	−0.0051 (10)	0.0005 (10)	−0.0130 (10)
C18A	0.0094 (13)	0.0361 (17)	0.0298 (16)	−0.0019 (11)	−0.0042 (11)	−0.0090 (13)
C19A	0.0155 (14)	0.0365 (17)	0.0346 (18)	0.0020 (12)	−0.0041 (12)	−0.0102 (14)
C20A	0.0225 (16)	0.0401 (18)	0.0318 (18)	−0.0013 (13)	−0.0058 (13)	−0.0002 (14)
C21A	0.0243 (16)	0.054 (2)	0.0238 (17)	0.0044 (14)	−0.0034 (13)	−0.0061 (15)
C22A	0.0223 (16)	0.046 (2)	0.0379 (19)	0.0052 (14)	−0.0034 (14)	−0.0172 (15)
C23A	0.0160 (14)	0.0363 (17)	0.0384 (18)	−0.0016 (12)	−0.0048 (13)	−0.0092 (14)
O1B	0.0129 (10)	0.0563 (14)	0.0323 (12)	0.0006 (9)	−0.0038 (9)	−0.0173 (10)
O2B	0.0137 (10)	0.0456 (13)	0.0300 (12)	−0.0013 (9)	0.0022 (8)	−0.0098 (10)
O3B	0.0141 (10)	0.0513 (14)	0.0253 (11)	0.0014 (9)	−0.0014 (8)	−0.0120 (10)
N1B	0.0141 (11)	0.0302 (13)	0.0276 (13)	−0.0003 (9)	−0.0019 (10)	−0.0069 (10)
C1B	0.0160 (14)	0.0375 (17)	0.0322 (17)	0.0010 (12)	0.0035 (13)	−0.0033 (13)
C2B	0.0215 (16)	0.0378 (18)	0.0302 (17)	0.0049 (13)	0.0010 (13)	−0.0049 (13)
C3B	0.0243 (16)	0.0305 (16)	0.0289 (17)	0.0017 (12)	−0.0069 (13)	−0.0079 (13)
C4B	0.0137 (14)	0.0305 (16)	0.0343 (17)	−0.0014 (11)	−0.0035 (12)	−0.0070 (13)
C5B	0.0138 (13)	0.0296 (16)	0.0296 (16)	0.0005 (11)	−0.0018 (12)	−0.0077 (12)
C6B	0.0184 (15)	0.0451 (19)	0.0331 (18)	0.0004 (13)	−0.0028 (13)	−0.0093 (14)
C7B	0.0216 (15)	0.0335 (17)	0.0354 (18)	−0.0010 (12)	−0.0033 (13)	−0.0105 (14)
C8B	0.0147 (14)	0.0395 (17)	0.0269 (16)	0.0001 (12)	−0.0006 (12)	−0.0099 (13)
C9B	0.0169 (14)	0.0324 (16)	0.0304 (17)	0.0026 (12)	−0.0060 (12)	−0.0112 (13)
C10B	0.0115 (13)	0.0299 (16)	0.0359 (17)	−0.0022 (11)	−0.0009 (12)	−0.0108 (13)
C11B	0.0190 (14)	0.0254 (15)	0.0278 (16)	−0.0014 (11)	0.0015 (12)	−0.0093 (12)
C12B	0.0145 (14)	0.0296 (15)	0.0285 (16)	−0.0006 (11)	−0.0016 (12)	−0.0102 (12)
C13B	0.0116 (13)	0.0364 (17)	0.0355 (18)	−0.0021 (12)	−0.0003 (12)	−0.0130 (13)
C14B	0.0170 (15)	0.051 (2)	0.0311 (18)	−0.0070 (14)	−0.0045 (13)	−0.0032 (15)
C15B	0.0157 (15)	0.060 (2)	0.0381 (19)	0.0010 (14)	−0.0078 (13)	−0.0147 (16)
C16B	0.0153 (15)	0.050 (2)	0.0388 (19)	−0.0040 (13)	0.0076 (13)	−0.0154 (15)
C17B	0.0139 (15)	0.061 (2)	0.0354 (19)	0.0030 (14)	−0.0036 (13)	−0.0176 (16)
S1B	0.0133 (3)	0.0366 (4)	0.0299 (4)	0.0012 (3)	−0.0015 (3)	−0.0090 (3)
O4B	0.0249 (12)	0.0504 (14)	0.0442 (14)	−0.0071 (10)	−0.0092 (10)	−0.0132 (11)
O5B	0.0397 (14)	0.0389 (13)	0.0370 (13)	0.0040 (10)	−0.0056 (10)	−0.0055 (10)
O6B	0.0162 (11)	0.0790 (18)	0.0346 (13)	0.0030 (11)	0.0001 (10)	−0.0177 (12)
C18B	0.0122 (13)	0.0318 (16)	0.0308 (16)	0.0032 (11)	−0.0013 (12)	−0.0109 (13)
C19B	0.0205 (15)	0.0369 (17)	0.0364 (18)	−0.0037 (12)	0.0019 (13)	−0.0143 (14)
C20B	0.0334 (18)	0.0330 (18)	0.0363 (19)	−0.0030 (14)	0.0055 (15)	−0.0064 (14)
C21B	0.0385 (19)	0.0379 (19)	0.0302 (18)	0.0074 (14)	−0.0064 (15)	−0.0097 (14)
C22B	0.0295 (18)	0.0363 (18)	0.044 (2)	0.0029 (14)	−0.0119 (15)	−0.0160 (15)
C23B	0.0188 (15)	0.0335 (17)	0.0368 (18)	−0.0015 (12)	−0.0022 (13)	−0.0110 (14)
O1W	0.0363 (14)	0.0420 (14)	0.0641 (17)	−0.0029 (11)	−0.0015 (12)	−0.0189 (12)
O2W	0.0273 (13)	0.0596 (16)	0.0433 (14)	0.0019 (11)	−0.0062 (10)	−0.0191 (12)

### Geometric parameters (Å, º)

**Table:**

O1A—C9A	1.365 (3)	O1B—C15B	1.429 (3)
O1A—C15A	1.435 (3)	O2B—C11B	1.354 (3)
O2A—C11A	1.358 (3)	O2B—C16B	1.431 (3)
O2A—C16A	1.433 (3)	O3B—C12B	1.363 (3)
O3A—C12A	1.372 (3)	O3B—C17B	1.439 (3)
O3A—C17A	1.431 (3)	N1B—C1B	1.347 (4)
N1A—C1A	1.347 (4)	N1B—C5B	1.370 (3)
N1A—C5A	1.377 (3)	N1B—C14B	1.483 (4)
N1A—C14A	1.478 (4)	C1B—C2B	1.356 (4)
C1A—C2A	1.372 (4)	C1B—H1BA	0.9300
C1A—H1AA	0.9300	C2B—C3B	1.394 (4)
C2A—C3A	1.393 (4)	C2B—H2BA	0.9300
C2A—H2AA	0.9300	C3B—C4B	1.374 (4)
C3A—C4A	1.371 (4)	C3B—H3BA	0.9300
C3A—H3AA	0.9300	C4B—C5B	1.400 (4)
C4A—C5A	1.391 (4)	C4B—H4BA	0.9300
C4A—H4AA	0.9300	C5B—C6B	1.441 (4)
C5A—C6A	1.430 (4)	C6B—C7B	1.352 (4)
C6A—C7A	1.358 (4)	C6B—H6BA	0.9300
C6A—H6AA	0.9300	C7B—C8B	1.433 (4)
C7A—C8A	1.451 (4)	C7B—H7BA	0.9300
C7A—H7AA	0.9300	C8B—C13B	1.408 (4)
C8A—C9A	1.409 (4)	C8B—C9B	1.415 (4)
C8A—C13A	1.418 (4)	C9B—C10B	1.386 (4)
C9A—C10A	1.384 (4)	C10B—C11B	1.390 (4)
C10A—C11A	1.383 (4)	C10B—H10B	0.9300
C10A—H10A	0.9300	C11B—C12B	1.417 (4)
C11A—C12A	1.414 (4)	C12B—C13B	1.371 (4)
C12A—C13A	1.368 (4)	C13B—H13B	0.9300
C13A—H13A	0.9300	C14B—H14D	0.9600
C14A—H14A	0.9600	C14B—H14E	0.9600
C14A—H14B	0.9600	C14B—H14F	0.9600
C14A—H14C	0.9600	C15B—H15D	0.9600
C15A—H15A	0.9600	C15B—H15E	0.9600
C15A—H15B	0.9600	C15B—H15F	0.9600
C15A—H15C	0.9600	C16B—H16D	0.9600
C16A—H16A	0.9600	C16B—H16E	0.9600
C16A—H16B	0.9600	C16B—H16F	0.9600
C16A—H16C	0.9600	C17B—H17D	0.9600
C17A—H17A	0.9600	C17B—H17E	0.9600
C17A—H17B	0.9600	C17B—H17F	0.9600
C17A—H17C	0.9600	S1B—O6B	1.445 (2)
S1A—O5AA	1.40 (4)	S1B—O5B	1.449 (2)
S1A—O4A	1.447 (2)	S1B—O4B	1.459 (2)
S1A—O5A	1.454 (2)	S1B—C18B	1.780 (3)
S1A—O6A	1.460 (2)	C18B—C19B	1.384 (4)
S1A—O6AA	1.49 (3)	C18B—C23B	1.389 (4)
S1A—O4AA	1.53 (4)	C19B—C20B	1.387 (5)
S1A—C18A	1.781 (3)	C19B—H19B	0.9300
C18A—C23A	1.385 (4)	C20B—C21B	1.384 (5)
C18A—C19A	1.391 (4)	C20B—H20B	0.9300
C19A—C20A	1.390 (4)	C21B—C22B	1.371 (5)
C19A—H19A	0.9300	C21B—H21B	0.9300
C20A—C21A	1.377 (5)	C22B—C23B	1.380 (4)
C20A—H20A	0.9300	C22B—H22B	0.9300
C21A—C22A	1.377 (5)	C23B—H23B	0.9300
C21A—H21A	0.9300	O1W—H1W1	1.0725
C22A—C23A	1.386 (4)	O1W—H2W1	0.9868
C22A—H22A	0.9300	O2W—H1W2	0.9268
C23A—H23A	0.9300	O2W—H2W2	0.9445
O1B—C9B	1.363 (3)		
			
C9A—O1A—C15A	117.9 (2)	C21A—C22A—C23A	119.7 (3)
C11A—O2A—C16A	116.5 (2)	C21A—C22A—H22A	120.2
C12A—O3A—C17A	116.5 (2)	C23A—C22A—H22A	120.2
C1A—N1A—C5A	121.4 (2)	C22A—C23A—C18A	120.2 (3)
C1A—N1A—C14A	117.5 (2)	C22A—C23A—H23A	119.9
C5A—N1A—C14A	121.1 (2)	C18A—C23A—H23A	119.9
N1A—C1A—C2A	122.0 (3)	C9B—O1B—C15B	117.9 (2)
N1A—C1A—H1AA	119.0	C11B—O2B—C16B	117.3 (2)
C2A—C1A—H1AA	119.0	C12B—O3B—C17B	116.7 (2)
C1A—C2A—C3A	118.1 (3)	C1B—N1B—C5B	122.1 (2)
C1A—C2A—H2AA	121.0	C1B—N1B—C14B	117.5 (2)
C3A—C2A—H2AA	121.0	C5B—N1B—C14B	120.4 (2)
C4A—C3A—C2A	119.7 (3)	N1B—C1B—C2B	121.7 (3)
C4A—C3A—H3AA	120.2	N1B—C1B—H1BA	119.1
C2A—C3A—H3AA	120.2	C2B—C1B—H1BA	119.1
C3A—C4A—C5A	121.7 (3)	C1B—C2B—C3B	118.5 (3)
C3A—C4A—H4AA	119.1	C1B—C2B—H2BA	120.7
C5A—C4A—H4AA	119.1	C3B—C2B—H2BA	120.7
N1A—C5A—C4A	117.2 (3)	C4B—C3B—C2B	119.6 (3)
N1A—C5A—C6A	119.6 (3)	C4B—C3B—H3BA	120.2
C4A—C5A—C6A	123.3 (3)	C2B—C3B—H3BA	120.2
C7A—C6A—C5A	123.0 (3)	C3B—C4B—C5B	121.1 (3)
C7A—C6A—H6AA	118.5	C3B—C4B—H4BA	119.4
C5A—C6A—H6AA	118.5	C5B—C4B—H4BA	119.4
C6A—C7A—C8A	126.4 (3)	N1B—C5B—C4B	116.9 (3)
C6A—C7A—H7AA	116.8	N1B—C5B—C6B	119.6 (3)
C8A—C7A—H7AA	116.8	C4B—C5B—C6B	123.5 (3)
C9A—C8A—C13A	117.8 (3)	C7B—C6B—C5B	123.0 (3)
C9A—C8A—C7A	118.1 (3)	C7B—C6B—H6BA	118.5
C13A—C8A—C7A	124.1 (3)	C5B—C6B—H6BA	118.5
O1A—C9A—C10A	123.1 (2)	C6B—C7B—C8B	126.7 (3)
O1A—C9A—C8A	115.8 (2)	C6B—C7B—H7BA	116.6
C10A—C9A—C8A	121.2 (3)	C8B—C7B—H7BA	116.6
C11A—C10A—C9A	119.9 (3)	C13B—C8B—C9B	117.6 (3)
C11A—C10A—H10A	120.0	C13B—C8B—C7B	123.6 (3)
C9A—C10A—H10A	120.0	C9B—C8B—C7B	118.8 (3)
O2A—C11A—C10A	124.7 (2)	O1B—C9B—C10B	123.3 (2)
O2A—C11A—C12A	115.1 (2)	O1B—C9B—C8B	115.3 (3)
C10A—C11A—C12A	120.2 (3)	C10B—C9B—C8B	121.4 (3)
C13A—C12A—O3A	125.7 (3)	C9B—C10B—C11B	119.3 (3)
C13A—C12A—C11A	119.7 (3)	C9B—C10B—H10B	120.3
O3A—C12A—C11A	114.6 (2)	C11B—C10B—H10B	120.3
C12A—C13A—C8A	121.2 (3)	O2B—C11B—C10B	124.8 (2)
C12A—C13A—H13A	119.4	O2B—C11B—C12B	114.6 (2)
C8A—C13A—H13A	119.4	C10B—C11B—C12B	120.6 (3)
N1A—C14A—H14A	109.5	O3B—C12B—C13B	126.0 (2)
N1A—C14A—H14B	109.5	O3B—C12B—C11B	114.9 (2)
H14A—C14A—H14B	109.5	C13B—C12B—C11B	119.1 (3)
N1A—C14A—H14C	109.5	C12B—C13B—C8B	121.9 (3)
H14A—C14A—H14C	109.5	C12B—C13B—H13B	119.0
H14B—C14A—H14C	109.5	C8B—C13B—H13B	119.0
O1A—C15A—H15A	109.5	N1B—C14B—H14D	109.5
O1A—C15A—H15B	109.5	N1B—C14B—H14E	109.5
H15A—C15A—H15B	109.5	H14D—C14B—H14E	109.5
O1A—C15A—H15C	109.5	N1B—C14B—H14F	109.5
H15A—C15A—H15C	109.5	H14D—C14B—H14F	109.5
H15B—C15A—H15C	109.5	H14E—C14B—H14F	109.5
O2A—C16A—H16A	109.5	O1B—C15B—H15D	109.5
O2A—C16A—H16B	109.5	O1B—C15B—H15E	109.5
H16A—C16A—H16B	109.5	H15D—C15B—H15E	109.5
O2A—C16A—H16C	109.5	O1B—C15B—H15F	109.5
H16A—C16A—H16C	109.5	H15D—C15B—H15F	109.5
H16B—C16A—H16C	109.5	H15E—C15B—H15F	109.5
O3A—C17A—H17A	109.5	O2B—C16B—H16D	109.5
O3A—C17A—H17B	109.5	O2B—C16B—H16E	109.5
H17A—C17A—H17B	109.5	H16D—C16B—H16E	109.5
O3A—C17A—H17C	109.5	O2B—C16B—H16F	109.5
H17A—C17A—H17C	109.5	H16D—C16B—H16F	109.5
H17B—C17A—H17C	109.5	H16E—C16B—H16F	109.5
O5AA—S1A—O4A	140.5 (17)	O3B—C17B—H17D	109.5
O5AA—S1A—O5A	51.4 (18)	O3B—C17B—H17E	109.5
O4A—S1A—O5A	113.11 (15)	H17D—C17B—H17E	109.5
O5AA—S1A—O6A	61.6 (18)	O3B—C17B—H17F	109.5
O4A—S1A—O6A	113.33 (14)	H17D—C17B—H17F	109.5
O5A—S1A—O6A	112.66 (14)	H17E—C17B—H17F	109.5
O5AA—S1A—O6AA	112 (2)	O6B—S1B—O5B	113.29 (15)
O4A—S1A—O6AA	61.6 (14)	O6B—S1B—O4B	113.51 (14)
O5A—S1A—O6AA	150.0 (13)	O5B—S1B—O4B	111.97 (14)
O6A—S1A—O6AA	54.4 (14)	O6B—S1B—C18B	105.69 (13)
O5AA—S1A—O4AA	118 (2)	O5B—S1B—C18B	105.38 (13)
O5A—S1A—O4AA	72.0 (15)	O4B—S1B—C18B	106.17 (14)
O6A—S1A—O4AA	148.6 (15)	C19B—C18B—C23B	119.8 (3)
O6AA—S1A—O4AA	105 (2)	C19B—C18B—S1B	120.6 (2)
O5AA—S1A—C18A	113.1 (17)	C23B—C18B—S1B	119.6 (2)
O4A—S1A—C18A	105.98 (14)	C18B—C19B—C20B	119.6 (3)
O5A—S1A—C18A	105.38 (14)	C18B—C19B—H19B	120.2
O6A—S1A—C18A	105.50 (13)	C20B—C19B—H19B	120.2
O6AA—S1A—C18A	104.4 (13)	C21B—C20B—C19B	120.5 (3)
O4AA—S1A—C18A	102.7 (14)	C21B—C20B—H20B	119.8
C23A—C18A—C19A	119.8 (3)	C19B—C20B—H20B	119.8
C23A—C18A—S1A	120.4 (2)	C22B—C21B—C20B	119.6 (3)
C19A—C18A—S1A	119.7 (2)	C22B—C21B—H21B	120.2
C20A—C19A—C18A	119.5 (3)	C20B—C21B—H21B	120.2
C20A—C19A—H19A	120.2	C21B—C22B—C23B	120.7 (3)
C18A—C19A—H19A	120.3	C21B—C22B—H22B	119.7
C21A—C20A—C19A	120.1 (3)	C23B—C22B—H22B	119.7
C21A—C20A—H20A	120.0	C22B—C23B—C18B	119.9 (3)
C19A—C20A—H20A	120.0	C22B—C23B—H23B	120.1
C20A—C21A—C22A	120.7 (3)	C18B—C23B—H23B	120.1
C20A—C21A—H21A	119.7	H1W1—O1W—H2W1	120.7
C22A—C21A—H21A	119.7	H1W2—O2W—H2W2	111.6
			
C5A—N1A—C1A—C2A	−1.0 (4)	C21A—C22A—C23A—C18A	0.4 (4)
C14A—N1A—C1A—C2A	−179.6 (3)	C19A—C18A—C23A—C22A	1.7 (4)
N1A—C1A—C2A—C3A	−0.1 (5)	S1A—C18A—C23A—C22A	−177.6 (2)
C1A—C2A—C3A—C4A	0.9 (5)	C5B—N1B—C1B—C2B	1.2 (4)
C2A—C3A—C4A—C5A	−0.6 (5)	C14B—N1B—C1B—C2B	−179.9 (3)
C1A—N1A—C5A—C4A	1.2 (4)	N1B—C1B—C2B—C3B	−0.3 (5)
C14A—N1A—C5A—C4A	179.8 (2)	C1B—C2B—C3B—C4B	−0.8 (4)
C1A—N1A—C5A—C6A	−179.3 (3)	C2B—C3B—C4B—C5B	0.9 (4)
C14A—N1A—C5A—C6A	−0.7 (4)	C1B—N1B—C5B—C4B	−1.0 (4)
C3A—C4A—C5A—N1A	−0.4 (4)	C14B—N1B—C5B—C4B	−179.8 (3)
C3A—C4A—C5A—C6A	−179.9 (3)	C1B—N1B—C5B—C6B	−178.2 (3)
N1A—C5A—C6A—C7A	−179.5 (3)	C14B—N1B—C5B—C6B	2.9 (4)
C4A—C5A—C6A—C7A	−0.1 (5)	C3B—C4B—C5B—N1B	−0.1 (4)
C5A—C6A—C7A—C8A	−179.4 (3)	C3B—C4B—C5B—C6B	177.0 (3)
C6A—C7A—C8A—C9A	−179.4 (3)	N1B—C5B—C6B—C7B	−169.2 (3)
C6A—C7A—C8A—C13A	0.5 (5)	C4B—C5B—C6B—C7B	13.8 (5)
C15A—O1A—C9A—C10A	5.2 (4)	C5B—C6B—C7B—C8B	175.8 (3)
C15A—O1A—C9A—C8A	−176.0 (3)	C6B—C7B—C8B—C13B	−4.4 (5)
C13A—C8A—C9A—O1A	179.8 (3)	C6B—C7B—C8B—C9B	174.8 (3)
C7A—C8A—C9A—O1A	−0.2 (4)	C15B—O1B—C9B—C10B	−2.0 (4)
C13A—C8A—C9A—C10A	−1.3 (4)	C15B—O1B—C9B—C8B	178.2 (3)
C7A—C8A—C9A—C10A	178.6 (3)	C13B—C8B—C9B—O1B	176.6 (3)
O1A—C9A—C10A—C11A	179.9 (3)	C7B—C8B—C9B—O1B	−2.7 (4)
C8A—C9A—C10A—C11A	1.1 (4)	C13B—C8B—C9B—C10B	−3.2 (4)
C16A—O2A—C11A—C10A	−6.3 (4)	C7B—C8B—C9B—C10B	177.6 (3)
C16A—O2A—C11A—C12A	173.3 (3)	O1B—C9B—C10B—C11B	−177.8 (3)
C9A—C10A—C11A—O2A	179.7 (3)	C8B—C9B—C10B—C11B	2.0 (4)
C9A—C10A—C11A—C12A	0.2 (4)	C16B—O2B—C11B—C10B	−1.9 (4)
C17A—O3A—C12A—C13A	0.4 (4)	C16B—O2B—C11B—C12B	178.6 (2)
C17A—O3A—C12A—C11A	−179.1 (2)	C9B—C10B—C11B—O2B	−178.3 (3)
O2A—C11A—C12A—C13A	179.2 (3)	C9B—C10B—C11B—C12B	1.2 (4)
C10A—C11A—C12A—C13A	−1.2 (4)	C17B—O3B—C12B—C13B	4.9 (4)
O2A—C11A—C12A—O3A	−1.3 (4)	C17B—O3B—C12B—C11B	−176.0 (3)
C10A—C11A—C12A—O3A	178.3 (2)	O2B—C11B—C12B—O3B	−2.8 (4)
O3A—C12A—C13A—C8A	−178.5 (3)	C10B—C11B—C12B—O3B	177.7 (2)
C11A—C12A—C13A—C8A	1.0 (4)	O2B—C11B—C12B—C13B	176.5 (3)
C9A—C8A—C13A—C12A	0.2 (4)	C10B—C11B—C12B—C13B	−3.1 (4)
C7A—C8A—C13A—C12A	−179.7 (3)	O3B—C12B—C13B—C8B	−179.1 (3)
O5AA—S1A—C18A—C23A	71.9 (19)	C11B—C12B—C13B—C8B	1.8 (4)
O4A—S1A—C18A—C23A	−102.4 (3)	C9B—C8B—C13B—C12B	1.2 (4)
O5A—S1A—C18A—C23A	17.8 (3)	C7B—C8B—C13B—C12B	−179.5 (3)
O6A—S1A—C18A—C23A	137.2 (2)	O6B—S1B—C18B—C19B	129.7 (2)
O6AA—S1A—C18A—C23A	−166.4 (14)	O5B—S1B—C18B—C19B	−110.1 (3)
O4AA—S1A—C18A—C23A	−56.8 (16)	O4B—S1B—C18B—C19B	8.8 (3)
O5AA—S1A—C18A—C19A	−107.5 (19)	O6B—S1B—C18B—C23B	−50.5 (3)
O4A—S1A—C18A—C19A	78.3 (3)	O5B—S1B—C18B—C23B	69.7 (3)
O5A—S1A—C18A—C19A	−161.6 (2)	O4B—S1B—C18B—C23B	−171.4 (2)
O6A—S1A—C18A—C19A	−42.2 (3)	C23B—C18B—C19B—C20B	−0.7 (4)
O6AA—S1A—C18A—C19A	14.2 (14)	S1B—C18B—C19B—C20B	179.1 (2)
O4AA—S1A—C18A—C19A	123.9 (16)	C18B—C19B—C20B—C21B	−0.3 (5)
C23A—C18A—C19A—C20A	−2.5 (4)	C19B—C20B—C21B—C22B	1.3 (5)
S1A—C18A—C19A—C20A	176.8 (2)	C20B—C21B—C22B—C23B	−1.4 (5)
C18A—C19A—C20A—C21A	1.2 (4)	C21B—C22B—C23B—C18B	0.4 (5)
C19A—C20A—C21A—C22A	0.9 (5)	C19B—C18B—C23B—C22B	0.7 (4)
C20A—C21A—C22A—C23A	−1.7 (5)	S1B—C18B—C23B—C22B	−179.1 (2)

### Hydrogen-bond geometry (Å, º)

*Cg*_5_ and *Cg*_6_ are the centroids of the C18A–C23A and C18B–C23B rings, respectively.

**Table:**

*D*—H···*A*	*D*—H	H···*A*	*D*···*A*	*D*—H···*A*
O1*W*—H1*W*1···O5*B*	1.07	1.88	2.919 (3)	161
O1*W*—H2*W*1···O6*A*	0.99	1.87	2.843 (3)	169
O2*W*—H1*W*2···O5*A*^i^	0.93	1.98	2.894 (3)	169
O2*W*—H2*W*2···O4*B*	0.94	1.97	2.884 (4)	163
C1*A*—H1*AA*···O4*A*^ii^	0.93	2.24	3.159 (4)	169
C4*A*—H4*AA*···O6*A*	0.93	2.51	3.394 (3)	160
C1*B*—H1*BA*···O6*B*^iii^	0.93	2.34	3.216 (4)	157
C4*B*—H4*BA*···O4*B*^iv^	0.93	2.39	3.198 (3)	145
C6*B*—H6*BA*···O5*B*^v^	0.93	2.54	3.419 (4)	158
C14*B*—H14*D*···O6*B*^iii^	0.96	2.39	3.307 (4)	160
C14*B*—H14*F*···O5*B*^v^	0.96	2.51	3.331 (4)	143
C19*A*—H19*A*···O1*W*	0.93	2.59	3.477 (4)	160
C20*A*—H20*A*···O2*B*^vi^	0.93	2.60	3.393 (5)	144
C3*A*—H3*AA*···*Cg*5	0.93	2.78	3.688 (3)	164
C16*A*—H16*C*···*Cg*6^iv^	0.96	2.79	3.453 (4)	127

Symmetry codes: (i) *x*, *y*+1, *z*; (ii) *x*+1, *y*, *z*; (iii) −*x*, −*y*+1, −*z*+1; (iv) −*x*+1, −*y*+1, −*z*+1; (v) *x*−1, *y*, *z*; (vi) −*x*+1, −*y*+1, −*z*.
